# The Transmembrane Morphogenesis Protein gp1 of Filamentous Phages Contains Walker A and Walker B Motifs Essential for Phage Assembly

**DOI:** 10.3390/v9040073

**Published:** 2017-04-09

**Authors:** Belinda Loh, Maximilian Haase, Lukas Mueller, Andreas Kuhn, Sebastian Leptihn

**Affiliations:** Sebastian Leptihn, Institute of Microbiology and Molecular Biology, University of Hohenheim, Garbenstrasse 30, 70599 Stuttgart, Germany; belinda.loh@uni-hohenheim.de (B.L.); Maximilian.Haase@uni-hohenheim.de (M.H.); lukas.mueller@uni-hohenheim.de (L.M.); andreas.kuhn@uni-hohenheim.de (A.K.)

**Keywords:** filamentous phage, M13, gp1, zonula occludens toxin (Zot), phage assembly, assembly complex, ATPase, membrane protein, molecular hinge, secretion, Walker motifs

## Abstract

In contrast to lytic phages, filamentous phages are assembled in the inner membrane and secreted across the bacterial envelope without killing the host. For assembly and extrusion of the phage across the host cell wall, filamentous phages code for membrane-embedded morphogenesis proteins. In the outer membrane of *Escherichia coli*, the protein gp4 forms a pore-like structure, while gp1 and gp11 form a complex in the inner membrane of the host. By comparing sequences with other filamentous phages, we identified putative Walker A and B motifs in gp1 with a conserved lysine in the Walker A motif (K14), and a glutamic and aspartic acid in the Walker B motif (D88, E89). In this work we demonstrate that both, Walker A and Walker B, are essential for phage production. The crucial role of these key residues suggests that gp1 might be a molecular motor driving phage assembly. We further identified essential residues for the function of the assembly complex. Mutations in three out of six cysteine residues abolish phage production. Similarly, two out of six conserved glycine residues are crucial for gp1 function. We hypothesise that the residues represent molecular hinges allowing domain movement for nucleotide binding and phage assembly.

## 1. Introduction

Filamentous phages, in particular M13, are well known for their broad applications in phage display technology or as nanotechnology tools [1,2,3,4]. Often overseen is their highly fascinating life cycle: a plasmid-like genome which codes for only eleven proteins altogether, that allow the infection of the host, reproduction, and assembly of the phage. This minimalistic design has fascinated researchers for decades. Most aspects of the infection, genome multiplication, and assembly have been investigated in detail [1,5,6,7,8,9]. However, some aspects are still not well understood, in particular how the membrane-embedded phage proteins, which are not part of the phage filament, allow the assembly and secretion of the phage. In the outer membrane of *Escherichia coli*, a pore-like protein gp4 (or g4p, p4, or gpIV), which has been structurally solved by cryo-electron microscopy, allows the secretion of an assembled phage [10]. Gp4 is part of a larger complex with phage proteins in the inner membrane that are products of *geneI*, called gp1 (or g1p, p1, gpI, or Zot, the latter known from the *Vibrio cholerae* phage CTXΦ). The M13 *geneI* (and that of most filamentous phages) displays an internal start codon which results in the production of an N-terminally truncated gp1 fragment called gp11 [11]. Both proteins form a complex in the inner membrane, with yet unknown stoichiometry, hereafter referred to as the assembly complex [12]. Gp11 displays a transmembrane (TM) segment, but lacks the large cytoplasmic domain which contains a putative adenosine triphosphatase (ATPase) domain [5,13,14]. Nevertheless, both gp1 and gp11 are essential for the production of phage progeny [13].

Previously, it was shown that ATP is required for the assembly of the filamentous phage f1 [15]. It was speculated that a sequence found in the N-terminal region of gp1 of most filamentous phages represents a Walker A motif which could allow the binding and hydrolysis of ATP concomitant with a conformational movement in the domain. However, ATPase activity of gp1 has never been demonstrated. A previous in vitro study showed that the homologues protein from a filamentous phage CTXΦ, Zot (zonula occludens toxin) does not exhibit ATPase activity, however, this could potentially be due to denaturing conditions during its chromatographic purification [16].

In the periplasm, the assembly complex interacts with gp4 that forms a pore in the outer membrane of *E. coli* [17]. The interaction between gp1-gp11 and gp4 has been shown to be the result of several charged residues that are also crucial for the production of phages [5,14]. In addition, the gp1-gp11 complex requires the host protein thioredoxin, which participates directly in assembly [15]. Indeed, host cells lacking thioredoxin do not allow filamentous phage production [18]. Surprisingly, it is not the cysteine-formation properties of thioredoxin that is needed, but, instead, a DNA handling property of the host protein is potentially required [19].

In this work, we performed in vivo complementations *in trans* using an *amberI* phage and an isopropyl β-D-1-thiogalactopyranoside (IPTG)-inducible plasmid to demonstrate that the protein gp1 from the M13 phage contains an essential lysine residue in the Walker A motif that—when mutated—abolishes phage production. We also identified a Walker B motif with conserved aspartic and glutamic acid residues that are essential for the production of phages, indicating that the gp1-gp11 complex is likely to be an ATPase. In addition, we could show that two conserved cysteine residues in the periplasm and the cytoplasmic cysteine residue at position 90 are essential for phage assembly. Furthermore, the two periplasmic cysteines potentially form a disulphide bridge. Lastly, we investigated the role of several conserved glycine residues that might potentially allow conformational changes between domains in a hinge-like function. From a total of six highly conserved glycines, two seem to play a major role, as their mutation abolishes phage production.

## 2. Materials and Methods

Molecular biology: QuikChange II Site-directed mutagenesis was performed following the company’s protocol (Agilent Technologies Inc., Santa Clara, CA, USA). The numbering follows the sequence of the M13 gp1.

Phage production: M13 Phage was plated on Luria-Bertani (LB) plates that were top-layered with *E. coli* mixed with LB agar (0.7% agar) and incubated at 37 °C overnight to develop plaques. A single plaque was inoculated in 1 mL of LB broth and incubated for 1 h at room temperature. The M13 phage culture was then added to a 4 mL exponentially growing culture of *E. coli* strain K37 or K38 that was grown in LB broth until an OD600 of 0.5; after inoculation, the culture was grown at 37 °C for 5 h. The bacteria-phage culture was separated by centrifugation with the supernatant constituting the phage stock. To determine phage titer, dilutions of the supernatant were made with LB broth and “spotted” on LB plates that were top-layered with *E. coli* mixed with LB agar (0.7% agar). After incubating at 37 °C overnight, plaques grown were counted, and the phage titer was calculated based on the dilution factor.

In vivo complementation: For the complementation *in trans*, we first conjugated the *E. coli* M15 strain (Qiagen, Hilden, Germany) with MC4100 carrying a F-plasmid that contains a tetracycline resistance. By selecting with kanamycin and tetracycline, only M15 F+ was obtained. This strain was transformed with the pQE60 plasmid (Qiagen) containing the *geneI* or *geneI* mutants under an IPTG-inducible promoter. The M15 cells were grown to an OD600 of 0.8 and mixed with LB top agar (containing 0.7% agar and 0.02 mM IPTG). First, serial dilutions (1:10 steps) of the phage culture were spotted, with 5 μL per spot, onto the M15 layered-plates and incubated at 37 °C overnight. Once the right concentration had been determined, which still allowed the counting of plaques, a volume of 100 μL of phage was mixed into the top agar together with 300 μL of M15 cells and 0.02 mM IPTG, to obtain a larger amount of countable plaques for precise statistical analyses. Each experiment was repeated three times with and without IPTG in the medium (final concentration 0.02 mM).

Test for cysteine bridge formation: Protein expression of an N-terminal hexa-histidine-gp1 was induced with 0.5 mM IPTG in *E. coli* M15 containing pQE60 plasmids coding for gp1-C30S, C90S, C146S, C256S, and wild-type, respectively. After 1 h of induction, cells were collected and resuspended in 200 mM Tris-HCl (pH 8.4). Iodoacetamide (Sigma-Aldrich Chemie GmbH, Munich, Germany) was then added to a final concentration of 100 mM and samples were incubated at 25 °C for 1 h, which leads to an alkylation of free cysteines. The sample was precipitated using three volumes of 100% acetone. Laemmli buffer (with or without beta-mercaptoethanol) was then added to the sample and heated (or not) at 95 °C for 10 min before the proteins were separated on a 10% sodium dodecyl sulfate-polyacrylamide (SDS-PAGE) gel. In order to analyse the migration behaviour, Western blot analysis with anti-His antibodies (Sigma-Aldrich Chemie GmbH) was performed.

## 3. Results

### 3.1. Wild-Type GeneI Complements an AmberI Phage in Trans

**Figure 1:**
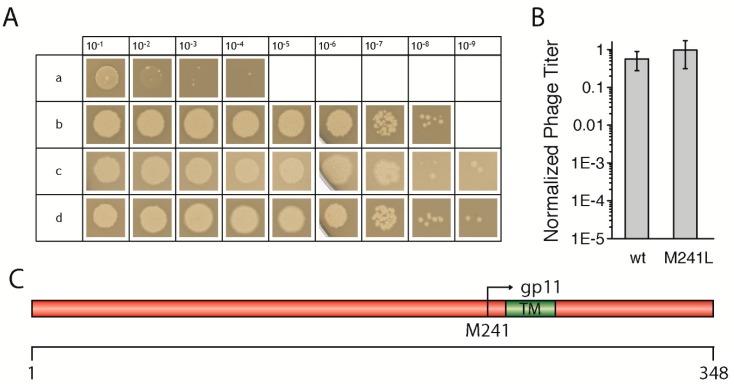
(**A**) Representative “spot assay” in which serial dilutions of phages were spotted on a bacterial lawn in order to analyse the amount of complementation compared to the wild-type. As controls, *amberI* phages were always spotted on the non-suppressor strain *Escherichia coli* K38 (a) and the *amber* suppressor strain *E. coli* K37 (b) to determine the phage titer of the solution. For complementation *in trans*, the *E. coli* strain M15 was used (c, d). Plasmid-encoded wild-type gp1 showed plaques to about the same dilution level as *E. coli* K37 (c), demonstrating that the induction of the protein *in trans* is able to fully complement the *amber* gene. A mutation of the internal start codon to leucine (M241L) had no effect, since gp11 was still made in the *amber* phage (d). (**B**) Quantification of phage titer from in vivo complementations of *geneI*. The amount of phages produced using plasmid-encoded wild-type gp1 is near-identical to the phage titer determined by using the *amber*-suppressor strain *E. coli* K37. Plasmid-encoded gp1 was normalised to 1, plotted on a logarithmic scale, and compared with the gp1 mutant M241L. The M241L mutation has no effect in this assay, although only gp1 is produced from plasmid-derived mRNA. The *amberI* phage still allows the expression of gp11, thus allowing full complementation of the gene, as both gene products are essential for the functionality of the protein [13]. (**C**) Schematic representation of the protein gp1 with its internal open reading frame (ORF), gp11, at methionine at position 241. The transmembrane (TM) segment is depicted in green. Numbers below indicate amino acid residue positions.

To test the effect of mutations in *geneI*, we first had to establish an in vivo complementation assay *in trans* using an IPTG-inducible plasmid coding for the gene. After testing several promoters and host strains, we chose the combination of the pQE60 vector which contains a T5 promoter recognized by the host’s own polymerase, together with the *E. coli* strain M15, which gave the most reproducible results. The non-suppressor *E. coli* strains K38, as well as M15, both produced similar levels of the *amberI* phage, however, using *E. coli* M15 led to the production of larger and clearer plaques, and was therefore used in all experiments (Figure 1A). Briefly, bacterial cells containing the plasmid coding for a wild-type *geneI* were grown, mixed with top agar, and then added on top of LB plates containing IPTG. After solidification of the agar, serial dilutions of the *amberI* phage were “spotted” onto the plates and incubated overnight, to be counted in the morning. Controls with the *amber*-suppressor strain *E. coli* K37 were performed for the determination of the phage titer of the phage stock solution. For precise statistical analyses, the phage dilutions were mixed with the host strain in the top agar on whole plates and counted in three independent experiments.

The *amberI* phage used contains an *amber* stop codon following codon 22 (Q23am). Thus, in a non-suppressor strain such as M15 or K38, only a small gp1-fragment is produced which is not functional (Figure 1Aa). Wild-type *geneI* codes for gp1, but also contains an in-frame start codon, resulting in an N-terminally truncated version of gp1, called gp11. When M15 was transformed with a plasmid coding for the wild-type *geneI*, phages were produced in amounts that were almost identical to the phage titer as determined when the *amber*-suppressor strain K37 was used (Figure 1Ac,B). Plasmid-encoded *geneI* complemented to wild-type levels at an IPTG concentration of 0.02 mM, whereas leaky expression of *geneI* without IPTG still allowed phages to be produced, albeit to a lesser extent (data not shown). Similarly, a *geneI* coding for gp1-M241L complemented to approximately the same level as wild-type *geneI* (Figure 1Ad,B). In the gp1-M241L construct, the internal start codon in position 241, coding for gp11, is replaced by a leucine, abolishing the expression of gp11 (Figure 1C). Although gp11 was shown to be essential for the production of the f1 phage, a close relative of M13 [13], the *amberI* phage still allowed the expression of the internal open reading frame coding for gp11. Therefore, the plasmid construct coding for g1p-M241L was able to fully complement the *amberI* phage. The results demonstrate the validity of the established in vivo complementation assay *in trans*, allowing subsequent tests to investigate the influence of various mutations in *geneI* on the morphogenesis of phages. The mutations were created based on sequence comparisons of filamentous phages (Figure S1) in order to understand the functional role, as well as structural aspects of the morphogenesis proteins gp1 and gp11.

### 3.2. Lys14 in a Putative Walker A Motif is Essential for Phage Production

**Figure 2:**
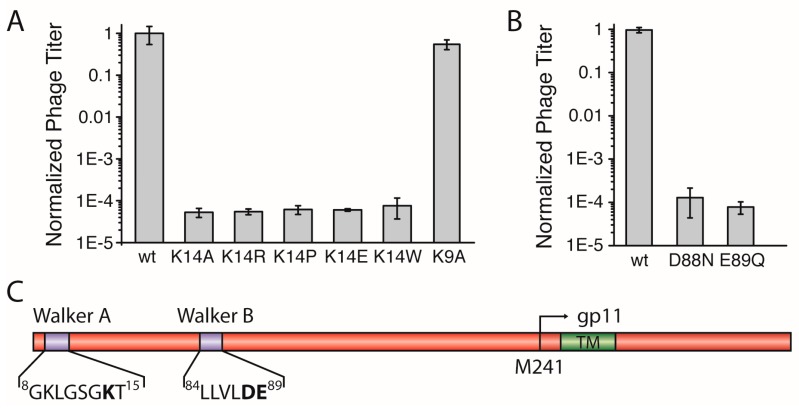
(**A**) Quantified phage titer in in vivo complementation assays of *geneI* mutants in the Walker A motif. The amount of phages produced using plasmid-encoded wild-type gp1 was normalised to 1 and compared with gp1 mutants, plotted on a logarithmic scale. The codon for lysine residue in position 14 (K14) in the putative Walker A motif was mutated to alanine (K14A), arginine (K14R), proline (K14P), glutamic acid (K14E), and tryptophan (K14W). None of the mutations can substitute for the lysine, indicating a crucial catalytic role of the residue contained within the motif. In addition, the effect of the mutation of lysine in position 9 to alanine (K9A) is shown on the right. The mutation in this position does not influence phage production. (**B**) In vivo complementation of *geneI* mutants in the putative Walker B motif. A mutation of the codon in position 88 from aspartate to asparagine (D88N) abolishes phage production. Similarly, the exchange of the residue glutamate to glutamine in position 89 (E89Q) results in the loss of phage production. (**C**) Schematic representation of the gp1 protein with its internal ORF g11p. The two Walker motifs, Walker A and Walker B, are shown in purple with the respective sequences below. Numbers indicate amino acid residues. Key residues crucial for phage production are in bold. The transmembrane (TM) segment is depicted in green.

The N-terminal region of M13 gp1 displays a sequence with a putative Walker A motif that is crucial for the hydrolysis of ATP in functional ATPases [20]. The motif, conserved among most filamentous phages with the consensus sequence: GXXXXGKT/S, contains a lysine which is found in position 14 in the M13 gp1 (sequence: ^8^GKLGSGKT^15^, Figure 2C, Figure S2). To investigate whether the residue is essential for the formation of phages, we mutated K14 to alanine (gp1-K14A) and tested the effect of the mutation in the above-described complementation assay *in trans* using an IPTG-inducible plasmid together with a M13 *amberI* phage.

Using the plasmid-encoded gp1-K14A, plaques were observed to a dilution level of 10^5^, about ten times more than the *amberI* phage without plasmid. In addition to phages produced due to reversion and transmission, this background observation can be attributed to the formation of functional phages from recombination events. Statistical analyses in which complementation of the plasmid-encoded wild-type gp1 was compared to the lysine mutant showed that gp1-K14A allows the formation of ten-thousand times less plaques that the wild-type, demonstrating the crucial role of the residue for the function of the protein (Figure 2A).

Lysine has previously been shown to play an essential role in the binding and hydrolysis of ATP in a Walker A motif [21]. However, whether the charge of lysine is the only factor required for phage production is unclear. Therefore, we mutated the amino acid to arginine. Again, complementation was not observed, as plaques were only formed to about the same concentration of that of g1p-K14A. The statistical analysis of the number of plaques from three independent experiments show that no difference is exhibited whether K14 is mutated to an Ala or an Arg (Figure 2A). In addition, three other mutations at position 14, including proline, tryptophan, and the negatively charged glutamate, resulted in a low amount of plaques being formed, with numbers almost identical to the gp1-K14A (Figure 2A). To ensure that the mutation did not abolish expression of the proteins, two such mutations (K14A and K14R) were introduced in a plasmid coding for an N-terminal hexa-Histidine fusion protein due to the lack of an antibody specific to gp1. After expression, the wild-type and the mutant proteins were analysed by immunoblotting. Both gp1-K14A and gp1-K14R were expressed at similar levels as the wild-type gp1, indicating that the lack of phage production is not due to the absence of the gp1 protein, but rather due to the mutation in Walker A (Figure S4A).

This finding demonstrates the crucial role of K14 for the production of phages. The coordination of ATP in a Walker A requires specifically a lysine that is accurately positioned in the motif [22], as the similarly charged residue arginine does not preserve the functionality of the motif. Therefore, these results strongly support the hypothesis that gp1 contains a Walker A motif important for ATP-hydrolysis.

Aside from the catalytically important lysine residue in position 14, a second lysine can be found in position 9 of gp1 (Figure 2C). In the cell-division protein MinD, a Walker A motif similar to the one in gp1 can be found with a lysine preceding the catalytic K14. This kind of Walker sequence was also termed a “deviant Walker A motif” [22]. In MinD, this residue is important for dimerisation of ATPase subunits [23]. If such a function is important for gp1, and is also mediated by K9 in gp1, we would expect a reduced amount of phages, as subunit interactions are often crucial for the proper function of protein complexes. However, the in vivo complementation assay showed that gp1-K9A produced similar amounts of phages as wild-type gp1 (Figure 2A). The results indicate that K9 contained within the Walker A motif is not crucial for protein function, and thus seems not to play an important role for subunit interaction.

### 3.3. Asp88 and Glu89 in a Putative Walker B Motif are Essential for the Formation of Phage Progeny

For the hydrolysis of ATP both Walker motifs, A and B, are essential. In the previous section, we described the identification of the Walker A motif with the catalytic residue K14 in gp1. The Walker B motif is less distinct and contains two catalytic residues, an aspartic acid residue followed by a glutamic acid, both preceded by a stretch of four hydrophobic amino acids [24]. We were able to identify such a motif in positions ^84^LLVLDE^89^ (Figure 2C, Figure S2) and constructed two *geneI* mutants coding for gp1-D88N and gp1-E89Q, respectively, to test in the complementation assay. Both mutations created chemically similar environments, but lacked the charge and do not represent residues found in functional Walker B motifs. In the complementation assays, neither of the mutants was able to complement and did not allow the production of phages (Figure 2B). Again, the expression of both gp1 mutants (D88N and E89Q) was tested as a hexa-Histidine fusion protein. Immunoblotting confirmed that both mutants were produced at similar levels as wild-type gp1 (Figure S4B). The analyses of three independent complementation experiments showed only levels that are attributable to transmission, reversion, and recombination events. These observations clearly demonstrate the critical role of D88 and E89 within the motif, which is therefore likely to represent a functional Walker B motif.

### 3.4. The Two Periplasmic Cysteine Residues 332 and 347 and the Cytoplasmic Cysteine Residue 90 Are Essential for Phage Production

**Figure 3:**
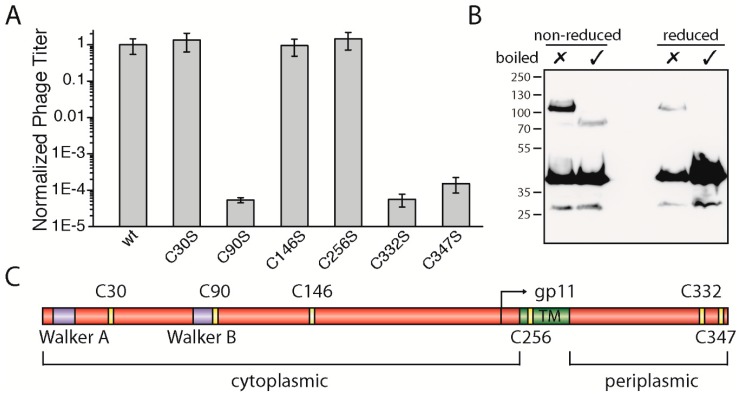
(**A**) Quantified phage titer in in vivo complementation assays of *geneI* cysteine mutants. The amount of phages produced using plasmid-encoded wild-type gp1 was normalized to 1 and compared with gp1 mutants, plotted on a logarithmic scale. While the substitution of cysteine with the chemically similar residue serine had no effect on protein function in position 30 (C30S), 146 (C146S), and 256 (C256S), mutations in positions 90 (C90S), 332 (C332S), and 347 (C347S) severely affected the production of phages. (**B**) Western blot analysis showing the migration pattern of His-tagged gp1 quadruple mutant (C30S, C90S, C146S, C256S) under reducing and non-reducing conditions. Samples were either not boiled (✗) or boiled (✓). (**C**) Schematic representation of the protein gp1 with its internal ORF gp11. The two Walker motifs, Walker A and Walker B, are shown in purple, the TM in green, and the cysteine residues are depicted in yellow. Numbers refer to the residue of the cysteines within gp1/gp11.

The gp1 sequences of most filamentous phages display several highly conserved residues such as the above-described lysine in Walker A and the catalytic aspartic-glutamic acid residues in Walker B. Among the conserved residues, many cysteine residues are observed—specifically six altogether (Figure S3). According to bioinformatic tools that predict the position of a transmembrane helix between residues 254 and 270 in M13 gp1, there are three cysteines found in the cytoplasm: one in the membrane and two on the periplasmic side (Figure 3C). Among the filamentous phages, four cysteines (C90, C146, C332, and C347) are highly conserved and two to a lesser degree (C30 and C256).

To study the role of the cysteines, we mutated each residue to the chemically similar serine and performed complementation assays as described above. While residue C30 and C146 in the cytoplasm as well as residue C256 in the membrane did not affect phage production, two cysteine residues (gp1-C332S and gp1-C347S) located in the periplasm were found to be necessary for phage production. When mutated to serine, neither gp1-C332S nor gp1-C347S complemented in the in vivo assay (Figure 3A). These two residues found in the periplasm could, due to the oxidative environment, form disulphide bridges. Aside from intramolecular bonds, gp1 could form cysteine bridges between gp1 subunits or other proteins. Hence, to test whether gp1 forms disulphide bonded multimers via C332 and C347, we constructed a gp1 mutant which has all other cysteines mutated to serine, except the two periplasmic cysteines. This construct was over-expressed as a hexa-histidine N-terminal fusion protein in *E. coli*, and its migration behaviour was analysed under reducing and non-reducing conditions by immunoblotting. If gp1 monomers formed dimers or would associate with host proteins via cysteine residues, higher molecular weight species would be observed under non-reducing conditions. Indeed, without boiling and under non-reducing conditions, a band was observed at a higher molecular weight (ca. 110 kDa), suggesting that a dimer was formed via cysteine bridges (Figure 3B). Upon heating to 95 °C, a band at around 80 kDa became visible while the higher band disappeared. As membrane proteins often show a higher migration behaviour when not being fully denatured [25,26], we concluded that both bands are produced by the same protein (a gp1 dimer), with one being denatured and the other one being partially folded. This hypothesis was further confirmed by the observation that a faint band at around 110 kDa was observed when the sample was not boiled but reduced. Since cysteine bridges are destroyed in this sample, the gp1 dimer is likely to be stabilized by further interactions such as hydrophobic forces. The band disappears when the sample was heated as well as reduced; now only monomeric gp1 can be detected.

Among the cytoplasmic cysteine residues, residue 90 (gp1-C90S) was the only residue which resulted in the loss of phage production (Figure 3A). Gp1-C30S, gp1-C146S, and gp1-C256S complemented to wild-type gp1 levels, while plaques were only observed to a 10^5^ dilution in the case of gp1-C90S, similar to *E. coli* K38 without any plasmid. Statistical analyses of whole plates and a count of plaques show that phages are produced ten-thousand times less in comparison to the plasmid encoded wild-type gp1. One possible explanation why C90 is essential for the function of the gp1 complex is the formation of a disulphide bond. Cytoplasmic cysteine bridges are rare due to the reducing environment of the cytoplasm, but do exist in *E. coli* [27]. The C90 residue has no “partner” to form an intramolecular cysteine bridge, and thus could only form a disulphide bond between the subunits or potentially with another, yet unknown host protein. Like the periplasmic cysteines described above, we analysed the migration behaviour of gp1-C90 via immunoblotting. However, the migration profile of gp1-C90S was similar to those of the control, indicating that C90 probably does not participate in the formation of disulphide bridges, and more studies are needed to understand its role in phage formation (data not shown).

### 3.5. A Potential Hinge-Like Function of Gly29 and Gly118 Might Allow Conformational Changes in the gp1-gp11 Complex for Nucleotide Binding

**Figure 4:**
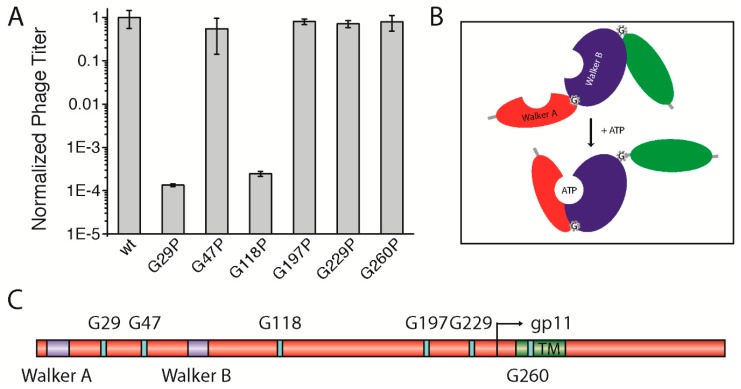
(**A**) Quantified phage titer in in vivo complementation assays of *geneI* glycine mutants. The amount of phages produced using plasmid-encoded wild-type gp1 was normalised to 1 and compared with gp1 mutants, plotted on a logarithmic scale. While the drastic structural substitution from glycine to proline had no effect on protein function in position 229 (G229P), 260 (G260P), 47 (G47P), and 197 (G197P), mutations in positions 29 (G29P) and 118 (G118P) severely affected the production of phages. Statistical analysis shown is a result of three independent experiments. (**B**) Model of gp1 illustrating the possible function of G29 and G118 in mediating conformational change. (**C**) Schematic representation of the gp1 protein with its internal ORF g11p. The two Walker motifs, Walker A and Walker B, are shown in purple, the TM in green, and the conserved glycine residues are depicted in light blue. The numbers refer to the glycine residues within gp1/gp11.

As the smallest amino acid, glycine has a special role in the structure of proteins; being highly flexible, the residue is often found in bends and turns, and can serve as a molecular hinge that allows two structural elements a large degree of conformational freedom in order to stay apart or to interact with each other. For this function, distinct glycine residues can mediate active or inactive protein conformations. Some membrane proteins, among them ion channels, contain highly conserved glycines that serve as a molecular hinge to allow the movement of the inner helix for gating [28]. Altogether, six highly conserved glycine residues were identified: one in between Walker A and Walker B motifs (G29), one following the Walker B motif (G47), three distributed along the cytoplasmic domain (G118, G197, and G229), and finally one within the predicted TM (G260) (Figure 4C, Figure S3). We hypothesized that one or more of the glycine residues might be important for the function of the assembly complex (e.g., for activating the ATPase upon binding of the phage DNA or for the “polymerization” of the coat proteins). Therefore, we mutated each residue to a proline, which creates a rigid kink in the polypeptide chain instead of the highly flexible glycine residue (gp1-G29P, gp1-G47P, gp1-G118P, gp1-G197P, gp1-G229P, and gp1-G260P). As the well conserved glycine residue G260 was also identified in the transmembrane region of the only distantly related *Vibrio cholerae* phage CTX-Φ, we tested gp1-G260P in the in vivo complementation assay first. Such an extreme structural rearrangement in the domain might result in the reduction of phage production. During translation, the ribosome incorporates proline always in its *trans* isomeric form [29]. Hence, we hypothesized that the complex should always be open/active or closed/inactive, with regards to the steric orientation of the domain within the complex. However, no change in phage production was observed, since the mutant complemented to approximately the same level as the plasmid-encoded wild-type did (Figure 4A).

Three other conserved glycine residues (G47, G197, and G229) were tested towards a potential hinge function. Again, similar amounts of phages were produced compared to the wild-type, which shows that these mutations had no influence on phage production (Figure 4A). Although being highly conserved, substituting glycines in these positions with the structure-distorting proline seemed to be tolerated by the gp1 protein, as they do not affect phage production. Next, we tested two other glycines, one positioned in between the two Walker motifs (G29), and another following Walker B (G118). A proline mutation had a dramatic effect on phage production in the case of gp1-G29P. Similar to mutations within the Walker motifs, only phages from reversion, transmission, and recombination events were produced (Figure 4A). Since the residue is found between the two Walker motifs that coordinate the binding and hydrolysis of nucleotides, the G29P mutation might inhibit the movement of the domains toward each other upon binding of ATP or lead to a distorted position of the two subdomains relative to each other (Figure 4B). Similarly, gp1-G118P did not allow the production of phages. As this glycine residue is found after the Walker B motif, it might allow movement of the nucleotide-binding domain relative to the DNA-binding domain that is yet to be identified. As the introduction of prolines can have dramatic effects on protein structure and expression, we tested whether the two mutants, which did not complement in the assay, were indeed expressed in the cells. Immunoblotting demonstrated that the proteins were produced to the same extent as was wild-type gp1 (Figure S4C).

## 4. Discussion

Bacteriophages contain fascinating nanomachines (e.g., to penetrate bacterial envelopes and to deliver their genome into the host or for the packaging of DNA into proheads) [30,31,32,33]. In this work, we characterized a membrane-embedded molecular motor of the non-lytic filamentous phage M13, products of *geneI* that function as a phage assembly complex. We mainly used in vivo complementation assays and performed a series of single-point mutations to study the role of conserved amino acids.

The most important finding of our work was the identification of the nucleotide binding motifs Walker A and Walker B. We could show that a putative Walker A motif with a key lysine residue is indeed crucial for the function of the complex. The K14 could not be replaced by the chemically similar aspartate, nor other chemically unrelated amino acids. In addition, we identified two key amino acids in a putative Walker B motif, D88 and E89, which suggests catalytic roles of the residues. These results indicate that the filamentous bacteriophage assembly complex is an ATP powered machine that assembles the phage in the inner membrane of the host.

Further crucial residues were identified that probably contribute to folding and stability of the protein. Three of the six cysteines in gp1-gp11 are essential for the function of the protein. The residues could potentially form inter- or intramolecular cysteine bonds. Disulphide bonds are very important for folding and stability of many proteins. As the cytoplasm of *E. coli* is a reducing environment, cysteine bridges in cytoplasmic proteins or protein domains are rare. In the periplasm, however, the formation of such bonds is favoured due to the oxidative nature of the compartment. We identified two crucial cysteine residues in the gp1 domain that is found on the periplasmic side of the membrane. When mutated to the chemically related serine, a complete loss of phage production was observed. Since both C332 and C347 are in an oxidative environment, the residues could form a cysteine bridge either within one subunit of gp1 or between two subunits. Our results indicate that two gp1 monomers might interact with each other via disulphide bonds. However, our data also show that most of gp1 runs as a monomer on a non-reducing SDS-PAGE gel. Inefficient dimerization might be due to the experimental conditions, including overexpression in the absence of other phage proteins and the addition of iodoacetamide, without allowing much time for the formation of cysteine bonds. Therefore, the role of the periplasmic cysteines needs further experimental confirmation, such as mass spectrometry of the purified proteins gp1 and gp11.

The other essential cysteine that was identified to abolish phage production is the cytoplasmic residue C90. Although disulphide bridges are rare in the reducing environment of the cytosol, we tested the hypothesis of whether gp1 forms dimers with each other or with another (host) protein via the residue. However, an intermolecular cysteine bridge formation was not detected, which points to another role of C90 that remains to be elucidated.

Filamentous phages are highly conserved among the *Enterobacteria*, but sequences from less related phages also show distinct homologies in some regions, which allows the identification of conserved residues. We discovered several conserved glycine residues that could potentially form molecular hinges, allowing the assembly complex to transition between a passive and an active state, or allow the movement of subdomains (e.g., for nucleotide binding). While most residues, among them G47, G197, G229, and G260, tolerated a proline mutation, the introduction of the amino acid into position 29 as well as 118 completely abolished phage production. We hypothesize that the residue G29 allows the movement of the domains containing Walker A and Walker B. This hinge movement is impeded by the removal of the flexible glycine and the replacement of the kink-inducing proline. Such a movement might be crucial for the simultaneous binding of ATP by the two Walker motifs that then sandwich the nucleotide between the subunits. Alternatively, the positions of the two domains that are in close vicinity even prior to nucleotide binding are distorted by the introduction of the proline. A second residue, G118, was identified that did not result in a functional protein when mutated to a proline. This residue might allow a movement between the two domains which contain the Walker motifs with a third domain that might bind the DNA during assembly [34]. So far, the hypotheses remain unconfirmed, and only structural studies or distance-sensitive measurements, such as fluorescence resonance energy transfer (FRET)-spectroscopy, might elucidate the roles of residues G29 and G118.

To our knowledge, the assembly complex represents the smallest membrane-bound molecular motor known so far. In vitro characterization of functional and structural aspects could let us understand this simple machine on a molecular level. However, due to the toxicity of the protein to the cell, a simple over-expression and subsequent purification the protein has so far proven to be very difficult, as also previously reported in the literature [35].

## Supplementary Materials

The following are available online at www.mdpi.com/1999-4915/9/4/73/s1, Figure S1: Alignment of filamentous phage gp1 proteins, Figure S2: Alignment of filamentous phage gp1 Walker A and Walker B motifs, Figure S3: Conserved Glycine and Cysteine residues in the alignment of filamentous phage gp1 proteins. Figure S4: Protein expression test of His-tagged gp1 mutants and wild-type.
