# Supplementary material for: The Transmembrane Morphogenesis Protein gp1 of Filamentous Phages Contains Walker A and Walker B Motifs Essential for Phage Assembly
Source: Viruses. 2017 Apr 9;9(4):73. doi: 10.3390/v9040073 (PMC5408679; doi:10.3390/v9040073)

Suppl. Fig. 1: Protein sequence homology alignment of gp1 proteins from filamentous phages. M13 for Bacteriophage M13 g1p, uniprot number P03656. I2 for Bacteriophage I2-2 g1p, uniprot number P15418. IF1 for Bacteriophage If1 g1p, uniprot number O80299. fd for Bacteriophage fd g1p, uniprot number P03655. IKe for Bacteriophage IKe g1p, uniprot number P03658. f1 for Bacteriophage f1 g1p, uniprot number P03657. V_VJF for *Vibrio cholerae* phage VJF-Phi ORF422. XantoPhiLf for *Xanthomonas* phage phiLf, uniprot number O55247. PropionibacB5 for Bacteriophage phiB5 of *Propionibacterium freudenreichii*.

Suppl. Fig. 2: Protein sequence homology alignment of the N-terminal region of gp1 proteins from filamentous phages. The putative Walker A and Walker B motifs are illustrated by red boxes. The consensus Walker motifs are depicted above the respective motifs. X represents any amino acid while h represents hydrophobic residues. M13 for Bacteriophage M13 g1p, uniprot number P03656. I2 for Bacteriophage I2-2 g1p, uniprot number P15418. IF1 for Bacteriophage If1 g1p, uniprot number O80299. fd for Bacteriophage fd g1p, uniprot number P03655. IKe for Bacteriophage IKe g1p, uniprot number P03658. f1 for Bacteriophage f1 g1p, uniprot number P03657. V_VJF for *Vibrio cholerae* phage VJF-Phi ORF422. XantoPhiLf for *Xanthomonas* phage phiLf, uniprot number O55247. PropionibacB5 for Bacteriophage phiB5 of *Propionibacterium freudenreichii*.

Suppl. Fig. 3: Protein sequence homology alignment of gp1 proteins from filamentous phages. All cysteine residues of M13 are shown in yellow boxes and numbered above. The six tested glycine residues with putative hinge-like functions are boxed in green and numbered above. I2 for Bacteriophage I2-2 g1p, uniprot number P15418. IF1 for Bacteriophage If1 g1p, uniprot number O80299. fd for Bacteriophage fd g1p, uniprot

number P03655. IKe for Bacteriophage IKe g1p, uniprot number P03658. M13 for Bacteriophage M13 g1p, uniprot number P03656. f1 for Bacteriophage f1 g1p, uniprot number P03657.

Suppl. Fig. 4: Western blot analysis of His-tagged gp1 mutants and wild type (wt) expressed in the *E. coli* M15 strain used in complementation assays. Top panel depicts protein detected by α-His antibody. Lower panel depicts protein detected by α-OmpA antibody. The Outer membrane protein A, OmpA was used as an expression control. (A) Gp1 Walker A mutants K14A and K14R. (B) Gp1 Walker B mutants D88N and E89Q. (C) Gp1 glycine mutations G29P and G118P.

Supplementary Figure 1

```
M13              1 MA-VY------------FVTGKLGSGKTLVSVG-KIQDKIVAGCKIATNLD-LRLQNLPQ
I2               1 MA-VY------------VVTGKLGAGKTLVAVS-RIQRTLAKGGIVATNLN-LKLHHFPQ
IF1              1 MA-VY------------VVTGKLGAGKTLVAVG-KIQDKIVSGCRVATNLD-LRIHKLPR
fd               1 MA-VY------------FVTGKLGSGKTLVSVG-KIQDKIVAGCKIATNLD-LRLQNLPQ
IKe              1 MA-VY------------VVTGKLGAGKTLVAVS-RIQRTLAKGGIVATNLN-LKLHHFPQ
f1               1 MA-VY------------FVTGKLGSGKTLVSVG-KIQDKIVAGCKIATNLD-LRLQNLPQ
V_VJF            1 MASVY------------FVTGKLGSGKSLIAVS-RIRDALMRGVPVATNLN-INLKEM--
XantoPhiLf       1 ML-VF-------------NEGVPRAGKSYDAVKNHILPALKKGRRVFARLNGLRFDRIAK
PropionibacB5    1 MS-WQSKRDLRRRFGIVGYVGSNGSGKSLMAVL-DTMPTLEAGRQVLSTVR-LLDWENPR

M13             46 V-GRFAK-TPRVLRIPDKPSISDLLAIGRGND----SYDENKNGLLVLDECGTWFNTRSW
I2              46 V-GRYAK-QCRVMRIADKPTLEDLEAIGRGNL----SYDESKNGLIVLDECGTWFNSRNW
IF1             46 V-GIFAK-SPDVIRIPDKPSLDDLLAIGRGNN----SYDENKNGLLVLDECGTWFNSRSW
fd              46 V-GRFAK-TPRVLRIPDKPSISDLLAIGRGND----SYDENKNGLLVLDECGTWFNTRSW
IKe             46 V-GRYAK-QCRVMRIADKPTLEDLESIGRGNL----TYDESKNGLLVLDECGTWFNSRNW
f1              46 V-GRFAK-TPRVLRIPDKPSISDLLAIGRGND----SYDENKNGLLVLDECGTWFNTRSW
V_VJF           45 I-GRDKR-NTRLYRLPDKPTVEDIEILGYVNK----SYDTSKDGLIVLDECGTWFNSRTW
XantoPhiLf      47 HLGMAENDVQHLLVLVDTKDVSKLFACTQDESGKWCIPDEFKDALVVIDEVHEFYVNER-
PropionibacB5   58 P-CDDP--GCDLPGHPDHLAAHPLWVPLRELS----QMLDFEGGDILLDEVTGVASSRES

M13            100 NDKERQPII-DWFLHARKLGWDIIFLVQDLSIVDKQARSALAEHVVYCRRLDRITLP---
I2             100 SDKSRQPVI-DWFLHARKLGWDVIFIIQDISLMDKQAREALAEHVVYCRRLDKLNIP---
IF1            100 ADKERQSVI-NWFLHARKLGWDIIFLIQDLSIMDKQARVALAEHVVYCRRLDKITIP---
fd             100 NDKERQPII-DWFLHARKLGWDIIFLVQDLSIVDKQARSALAEHVVYCRRLDRITLP---
IKe            100 SDKSRQPVI-DWCLHARKLGWDIIFIIQDISLMDKQARDALAEHVVYCRRLDKLNIP---
f1             100 NDKERQPII-DWFLHARKLGWDIIFLVQDLSIVDKQARSALAENVVYCRRLDRITLP---
V_VJF           99 NDKNRQALL-DRFLHIRKLGWDVIFIVQNISMVDKQAREGLAEHVVHCKRLDRMQIP---
XantoPhiLf     106 --KPLAPAVENFWALLGQNGGDAVIMTQWINRLHSAVKAR-IEKKNTFQKLTAIGMK---
PropionibacB5  111 --MSLPPQIANMLVQLRRRDVVLRWTTPAWSRADLIIRET-TQAVVLCRGAMSKTLPGRV

M13            156 -----------------------------------------------------------F
I2             156 -----------------------------------------------------------I
IF1            156 -----------------------------------------------------------F
fd             156 -----------------------------------------------------------F
IKe            156 -----------------------------------------------------------I
f1             156 -----------------------------------------------------------F
V_VJF          155 -----------------------------------------------------------F
XantoPhiLf     160 -----------------------------------------------------------G
PropionibacB5  168 WPSHRLIMSRVVSAADATALSAGGQTAGRVSSLARSVIRLEQCPAIQAYDTLDRVLSVPV

M13            157 VGTLYSLITGS--KMPLPKLHVGVVKYG---------DSQLSPTVERWLYTGKNLYNAYD
I2             157 IGGLISVLSGG--RLPLPKVHFGIVKYG---------DNPQSLTVDKWIYTGTDLYAAYD
IF1            157 IGSIYSVITGS--KLPLPKVHVGIVKYG---------DSPQSMTVERWTYTGRDLYAAYD
fd             157 VGTLYSLVTGS--KMPLPKLHVGVVKYG---------DSQLSPTVERWLYTGKNLYNAYD
IKe            157 IGGLISVLSGG--RLPLPKVHFGIVKYG---------DNPQSLTVDKWVYTGTDLYAAYD
f1             157 VGTLYSLITGS--KMPLPKLHVGVVKYG---------DSQLSPTVERWLYTGKNLYNAYD
V_VJF          156 LSTIVWILTLGQLKIPMPKLHIGIVKYG---------DTINALTVDKWMLWGTDLYSSYD
XantoPhiLf     161 RYRVTYFHTTS--PGKFEKVGGQTLKYDPAIFPLYDGYAPGAENTEVYEEGGKNVWAAMA
PropionibacB5  228 AEGGRCVVCGG--RRSVPGCSCGD--D---------GDSC--------------------

M13            206 TKQAFSSNYD-SGVYSYLTPYLSHGRYFKPLNLGQKMKLTKIYLKKFSRVLCLAI----G
I2             206 TKQIFTSDRELSPPFCPVSPYYTHGIFAVKRDAKYYMRMTKIYFKKMNRVWLMASFLALG
IF1            206 TKQAFSDAYE-HSSFSYLTPYLSHGRYAVKRDATFYMRLTRIYLKKYSRVLCLFC----G
fd             206 TKQAFSSNYD-SGVYSYLTPYLSHGRYFKPLNLGQKMKLTKIYLKKFSRVLCLAI----G
IKe            206 TKQIFTSDREISPPYCPLSPYYTHGIFSVKRDAKYYMRMTKIYFKKMNRVFLMASFLALG
f1             206 TKQAFSSNYD-SGVYSYLTPYLSHGRYFKPLNLGQKMKLTKIYLKKFSRVLCLAI----G
V_VJF          207 TKQIFQNHYP-HGTYSVLPPWYIHGRYTVPYTARNIMRITKILFRKYSRVAMFAV----G
XantoPhiLf     219 VRAAIFLTLGGVGIYF-FMHYFTKDRADPNKPMASA--------SQTTKPTHVGA----G
PropionibacB5  255 ----------------------GTGRGAGPLGVARD------------------------
```

```
M13            261 FASAFTYSYITQPK--PEVK-----KVVS------------QTY-DFDKFTIDSSQRLNL
I2             266 AGVGFFYKSRQINEQLSNMPVASAQANTT--------KTDHTID-ELPRLSINSFAQMGY
IF1            261 FVSAFTYLSLSKPEATPQIKPVTT-QIIT------------SRY-KPSELRITTSYRMGN
fd             261 FASAFTYSYITQPK--PEVK-----KVVS------------QTY-DFDKFTIDSSQRLNL
IKe            266 AACGIFYKSQAYSNQLQHIQDNSKTSVIS--------KTDQSAE-ILPRLSINSYSQMGY
f1             261 FASAFTYSYITQPK--PEVK-----KVVS------------QTY-DFDKFTIDSSQRLNL
V_VFJ          262 AAFGAAFWHFTSPE--PELI-----QLVQAQNQMLTSESDHKLSELLGGFTISRYTALP-
XantoPhiLf     266 FANGAPSVPIQPPP--PDPL-----ADLTQEQ-----RYVAQLA-DKGRIRLSARARVGD
PropionibacB5  269 ------PSRAGRPV--TALR-----AVES------------TSS----------------

M13            301 SYR--YVFKDSK-GKLINSDD---LQKQGYSLTYIDLCTVSIKKGNSNEIVKC--N----
I2             317 DVN--VSFKDAK-GKIYYSFD---LMKSGYALDIKDSCHITLRKRNYIQQVTC--EG---
IF1            307 AVG--FEEMDAK-KQKIASDD---LIKDGFRMVYITPCSVELIKDGKHEKVT---C----
fd             301 SYR--YVFKDSK-GKLINSDD---LQKQGYSLTYIDLCTVSIKKGNSNEIVKC--N----
IKe            317 DVS--VTFKDAK-AKIYNSFD---LIKDGYRVDIKDACHVTIVKKSYIQQITC--EG---
f1             301 SYR--YVFKDSK-GKLINSDD---LQKQGYSLTYIDLCTVSIKKGNSNEIVKC--N----
V_VFJ          314 DAP--VTFELSNGAKRLTSYE---LQSMGFEIEPLSRCEIIIKSGAQNETIHC-------
XantoPhiLf     313 QDRAWIQWIDAS-NNVVEELDLSQLRALGYSVSVVTY-GVRLSAGK--HIMVATAWPWTA
PropionibacB5  288 --------------------------------------DLAVRLGKKSEAEGMNPP----

M13                ------------------------------------------------------------
I2                 ------------------------------------------------------------
IF1                ------------------------------------------------------------
fd                 ------------------------------------------------------------
IKe                ------------------------------------------------------------
f1                 ------------------------------------------------------------
V_VFJ              ------------------------------------------------------------
XantoPhiLf     369 PIREKDARLYNMAPDGSGGAAGVATAGSDGGGADRDQVRGGVIEYGPRTQGTFPDNKGYS
PropionibacB5  306 ------------------------------------------------------------

M13                ------------
I2                 ------------
IF1                ------------
fd                 ------------
IKe                ------------
f1                 ------------
V_VFJ              ------------
XantoPhiLf     429 SSTSTPATTLQM
PropionibacB5  306 ------ASA---
```

Supplementary Figure 2

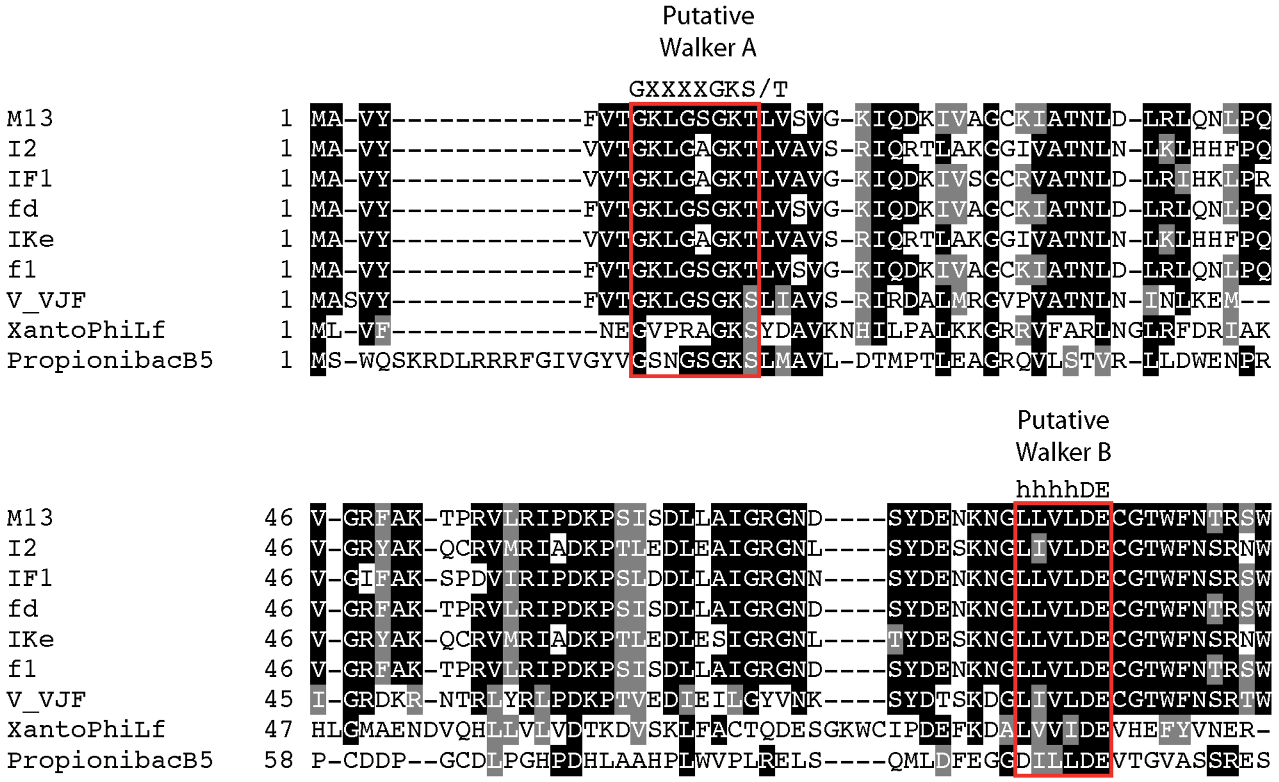

Supplementary Figure 3

```
                                                     Gly29 Cys30
M13            1 MA-VY------------FVTGKLGSGKTLVSVG-KIQDKIVAGCKIATNLD-LRLQNLPQ
I2             1 MA-VY------------VVTGKLGAGKTLVAVS-RIQRTLAKGGIVATNLN-LKLHHFPQ
IF1            1 MA-VY------------VVTGKLGAGKTLVAVG-KIQDKIVSGCRVATNLD-LRIHKLPR
fd             1 MA-VY------------FVTGKLGSGKTLVSVG-KIQDKIVAGCKIATNLD-LRLQNLPQ
IKe            1 MA-VY------------VVTGKLGAGKTLVAVS-RIQRTLAKGGIVATNLN-LKLHHFPQ
f1             1 MA-VY------------FVTGKLGSGKTLVSVG-KIQDKIVAGCKIATNLD-LRLQNLPQ
V_VJF          1 MASVY------------FVTGKLGSGKSLIAVS-RIRDALMRGVPVATNLN-INLKEM--
XantoPhiLf     1 ML-VF-------------NEGVPRAGKSYDAVKNHILPALKKGRRVFARLNGLRFDRIAK
PropionibacB5  1 MS-WQSKRDLRRRFGIVGYVGSNGSGKSLMAVL-DTMPTLEAGRQVLSTVR-LLDWENPR

                 Gly47                                       Cys90
M13           46 V-GRFAK-TPRVLRIPDKPSISDLLAIGRGND----SYDENKNGLLVLDECGTWFNTRSW
I2            46 V-GRYAK-QCRVMRIADKPTLEDLEAIGRGNL----SYDESKNGLIVLDECGTWFNSRNW
IF1           46 V-GIFAK-SPDVIRIPDKPSLDDLLAIGRGNN----SYDENKNGLLVLDECGTWFNSRSW
fd            46 V-GRFAK-TPRVLRIPDKPSISDLLAIGRGND----SYDENKNGLLVLDECGTWFNTRSW
IKe           46 V-GRYAK-QCRVMRIADKPTLEDLESIGRGNL----TYDESKNGLLVLDECGTWFNSRNW
f1            46 V-GRFAK-TPRVLRIPDKPSISDLLAIGRGND----SYDENKNGLLVLDECGTWFNTRSW
V_VJF         45 I-GRDKR-NTRLYRLPDKPTVEDIEILGYVNK----SYDTSKDGLIVLDECGTWFNSRTW
XantoPhiLf    47 HLGMAENDVQHLLVLVDTKDVSKLFACTQDESGKWCIPDEFKDALVVIDEVHEFYVNER-
PropionibacB5 58 P-CDDP--GCDLPGHPDHLAAHPLWVPLRELS----QMLDFEGGDILLDEVTGVASSRES

                                Gly118                     Cys146
M13           100 NDKERQPII-DWFLHARKLGWDIIFLVQDLSIVDKQARSALAEHVVYCRRLDRITLP---
I2            100 SDKSRQPVI-DWFLHARKLGWDVIFIIQDISLMDKQAREALAEHVVYCRRLDKLNIP---
IF1           100 ADKERQSVI-NWFLHARKLGWDIIFLIQDLSIMDKQARVALAEHVVYCRRLDKITIP---
fd            100 NDKERQPII-DWFLHARKLGWDIIFLVQDLSIVDKQARSALAEHVVYCRRLDRITLP---
IKe           100 SDKSRQPVI-DWCLHARKLGWDIIFIIQDISLMDKQARDALAEHVVYCRRLDKLNIP---
f1            100 NDKERQPII-DWFLHARKLGWDIIFLVQDLSIVDKQARSALAENVVYCRRLDRITLP---
V_VJF          99 NDKNRQALL-DRFLHIRKLGWDVIFIVQNISMVDKQAREGLAEHVVHCKRLDRMQIP---
XantoPhiLf    106 --KPLAPAVENFWALLGQNGGDAVIMTQWINRLHSAVKAR-IEKKNTFQKLTAIGMK---
PropionibacB5 111 --MSLPPQIANMLVQLRRRDVVLRWTTPAWSRADLIIRET-TQAVVLCRGAMSKTLPGRV

M13           156 -----------------------------------------------------------F
I2            156 -----------------------------------------------------------I
IF1           156 -----------------------------------------------------------F
fd            156 -----------------------------------------------------------F
IKe           156 -----------------------------------------------------------I
f1            156 -----------------------------------------------------------F
V_VJF         155 -----------------------------------------------------------F
XantoPhiLf    160 -----------------------------------------------------------G
PropionibacB5 168 WPSHRLIMSRVVSAADATALSAGGQTAGRVSSLARSVIRLEQCPAIQAYDTLDRVLSVPV

                                                                  Gly197
M13           157 VGTLYSLITGS--KMPLPKLHVGVVKYG---------DSQLSPTVERWLYTGKNLYNAYD
I2            157 IGGLISVLSGG--RLPLPKVHFGIVKYG---------DNPQSLTVDKWIYTGTDLYAAYD
IF1           157 IGSIYSVITGS--KLPLPKVHVGIVKYG---------DSPQSMTVERWTYTGRDLYAAYD
fd            157 VGTLYSLVTGS--KMPLPKLHVGVVKYG---------DSQLSPTVERWLYTGKNLYNAYD
IKe           157 IGGLISVLSGG--RLPLPKVHFGIVKYG---------DNPQSLTVDKWVYTGTDLYAAYD
f1            157 VGTLYSLITGS--KMPLPKLHVGVVKYG---------DSQLSPTVERWLYTGKNLYNAYD
V_VJF         156 LSTIVWILTLGQLKIPMPKLHIGIVKYG---------DTINALTVDKWMLWGTDLYSSYD
XantoPhiLf    161 RYRVTYFHTTS--PGKFEKVGGQTLKYDPAIFPLYDGYAPGAENTEVYEEGGKNVWAAMA
PropionibacB5 228 AEGGRCVVCGG--RRSVPGCSCGD--D---------GDSC--------------------

                                         Gly229                  Cys256  Gly260
M13           206 TKQAFSSNYD-SGVYSYLTPYLSHGRYFKPLNLGQKMKLTKIYLKKFSRVLCLAI----G
I2            206 TKQIFTSDRELSPPFCPVSPYYTHGIFAVKRDAKYYMRMTKIYFKKMNRVWLMASFLALG
IF1           206 TKQAFSDAYE-HSSFSYLTPYLSHGRYAVKRDATFYMRLTRIYLKKYSRVLCLFC----G
fd            206 TKQAFSSNYD-SGVYSYLTPYLSHGRYFKPLNLGQKMKLTKIYLKKFSRVLCLAI----G
IKe           206 TKQIFTSDREISPPYCPLSPYYTHGIFSVKRDAKYYMRMTKIYFKKMNRVFLMASFLALG
f1            206 TKQAFSSNYD-SGVYSYLTPYLSHGRYFKPLNLGQKMKLTKIYLKKFSRVLCLAI----G
V_VJF         207 TKQIFQNHYP-HGTYSVLPPWYIHGRYTVPYTARNIMRITKILFRKYSRVAMFAV----G
XantoPhiLf    219 VRAAIFLTLGGVGIYF-FMHYFTKDRADPNKPMASA--------SQTTKPTHVGA----G
PropionibacB5 255 ----------------------GTGRGAGPLGVARD------------------------
```

```
M13            261 FASAFTYSYITQPK--PEVK-----KVVS------------QTY-DFDKFTIDSSQRLNL
I2             266 AGVGFFYKSRQINEQLSNMPVASAQANTT---------KTDHTID-ELPRLSINSFAQMGY
IF1            261 FVSAFTYLSLSKPEATPQIKPVTT-QIIT-------------SRY-KPSELRITTSYRMGN
fd             261 FASAFTYSYITQPK--PEVK-----KVVS------------QTY-DFDKFTIDSSQRLNL
IKe            266 AACGIFYKSQAYSNQLQHIQDNSKTSVIS---------KTDQSAE-ILPRLSINSYSQMGY
f1             261 FASAFTYSYITQPK--PEVK-----KVVS------------QTY-DFDKFTIDSSQRLNL
V_VJF          262 AAFGAAFWHFTSPE--PELI-----QLVQAQNQMLTSESDHKLSELLGGFTISRYTALP-
XantoPhiLf     266 FANGAPSVPIQPPP--PDPL-----ADLTQEQ-----RYVAQLA-DKGRIRLSARARVGD
PropionibacB5  269 ------PSRAGRPV--TALR-----AVES------------TSS----------------

                                                      Cys332        Cys347
M13            301 SYR--YVFKDSK-GKLINSDD---LQKQGYSLTYIDLCTVSIKKGNSNEIVKC--N----
I2             317 DVN--VSFKDAK-GKIYYSFD---LMKSGYALDIKDSCHITLRKRNYIQQVTC--EG---
IF1            307 AVG--FEEMDAK-KQKIASDD---LIKDGFRMVYITPCSVELIKDGKHEKVT---C----
fd             301 SYR--YVFKDSK-GKLINSDD---LQKQGYSLTYIDLCTVSIKKGNSNEIVKC--N----
IKe            317 DVS--VTFKDAK-AKIYNSFD---LIKDGYRVDIKDACHVTIVKKSYIQQITC--EG---
f1             301 SYR--YVFKDSK-GKLINSDD---LQKQGYSLTYIDLCTVSIKKGNSNEIVKC--N----
V_VJF          314 DAP--VTFELSNGAKRLTSYE---LQSMGFEIEPLSRCEIIIKSGAQNETIHC-------
XantoPhiLf     313 QDRAWIQWIDAS-NNVVEELDLSQLRALGYSVSVVTY-GVRLSAGK--HIMVATAWPWTA
PropionibacB5  288 -------------------------------------DLAVRLGKKSEAEGMNPP----

M13                ------------------------------------------------------------
I2                 ------------------------------------------------------------
IF1                ------------------------------------------------------------
fd                 ------------------------------------------------------------
IKe                ------------------------------------------------------------
f1                 ------------------------------------------------------------
V_VJF              ------------------------------------------------------------
XantoPhiLf     369 PIREKDARLYNMAPDGSGGAAGVATAGSDGGGADRDQVRGGVIEYGPRTQGTFPDNKGYS
PropionibacB5  306 ------------------------------------------------------------

M13                ------------
I2                 ------------
IF1                ------------
fd                 ------------
IKe                ------------
f1                 ------------
V_VJF              ------------
XantoPhiLf     429 SSTSTPATTLQM
PropionibacB5  306 ------ASA---
```

Supplementary Figure 4

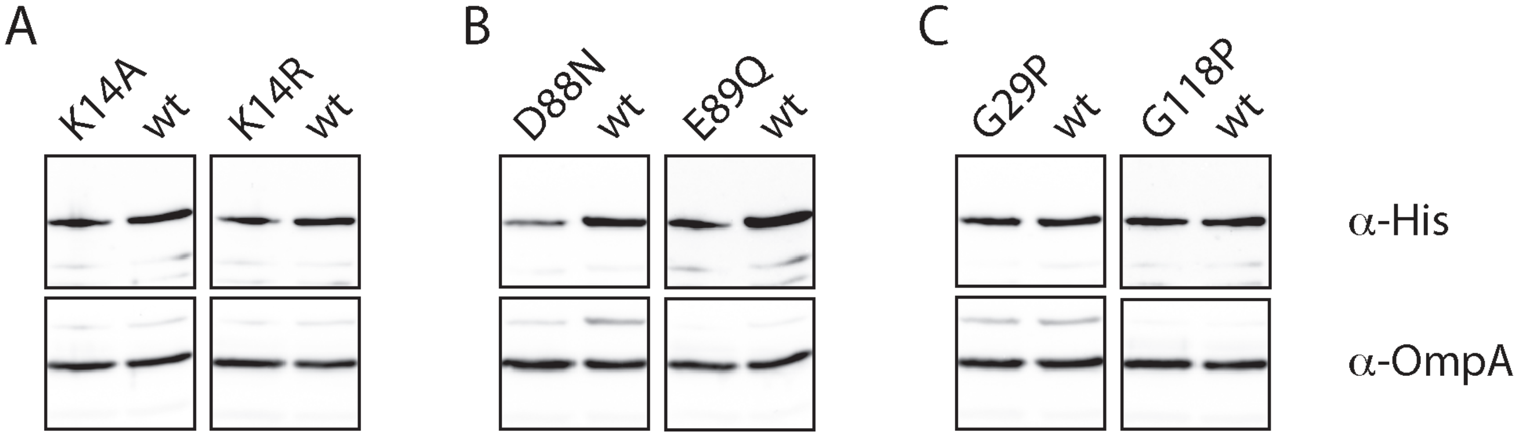

Supplement: Supplementary file 1 [file viruses-09-00073-s001.pdf]
